# Current challenges and future directions for engineering extracellular vesicles for heart, lung, blood and sleep diseases

**DOI:** 10.1002/jev2.12305

**Published:** 2023-02-12

**Authors:** Guoping Li, Tianji Chen, James Dahlman, Lola Eniola‐Adefeso, Ionita C. Ghiran, Peter Kurre, Wilbur A. Lam, Jennifer K. Lang, Eduardo Marbán, Pilar Martín, Stefan Momma, Malcolm Moos, Deborah J. Nelson, Robert L. Raffai, Xi Ren, Joost P. G. Sluijter, Shannon L. Stott, Gordana Vunjak‐Novakovic, Nykia D. Walker, Zhenjia Wang, Kenneth W. Witwer, Phillip C. Yang, Martha S. Lundberg, Margaret J. Ochocinska, Renee Wong, Guofei Zhou, Stephen Y. Chan, Saumya Das, Prithu Sundd

**Affiliations:** ^1^ Cardiovascular Research Center Massachusetts General Hospital and Harvard Medical School Boston Massachusetts USA; ^2^ Department of Pediatrics, College of Medicine University of Illinois at Chicago Chicago Illinois USA; ^3^ Department of Biomedical Engineering Georgia Institute of Technology and Emory University School of Medicine Atlanta Georgia USA; ^4^ Department of Biomedical Engineering University of Michigan Ann Arbor Michigan USA; ^5^ Department of Anesthesia and Pain Medicine Beth Israel Deaconess Medical Center, and Harvard Medical School Boston Massachusetts USA; ^6^ Children's Hospital of Philadelphia, Comprehensive Bone Marrow Failure Center, Perelman School of Medicine University of Pennsylvania Philadelphia Pennsylvania USA; ^7^ Wallace H. Coulter Department of Biomedical Engineering, Department of Pediatrics Emory School of Medicine Aflac Cancer and Blood Disorders Center of Children's Healthcare of Atlanta, Emory University and Georgia Institute of Technology Atlanta Georgia USA; ^8^ Department of Medicine, Division of Cardiology, Jacobs School of Medicine and Biomedical Sciences Veterans Affairs Western New York Healthcare System Buffalo New York USA; ^9^ Smidt Heart Institute Cedars‐Sinai Medical Center Los Angeles California USA; ^10^ Centro Nacional de Investigaciones Cardiovasculares (CNIC) Centro de Investigación Biomédica en Red de Enfermedades Cardiovasculares (CIBERCV) Madrid Spain; ^11^ Institute of Neurology (Edinger Institute) University Hospital Goethe University Frankfurt am Main Germany; ^12^ Division of Cellular and Gene Therapies, Office of Tissues and Advanced Therapies, Center for Biologics Evaluation and Research United States Food and Drug Administration Silver Spring Maryland USA; ^13^ Department of Pharmacological and Physiological Sciences The University of Chicago Chicago Illinois USA; ^14^ Department of Veterans Affairs, Surgical Service (112G) San Francisco VA Medical Center San Francisco California USA; ^15^ Department of Surgery, Division of Vascular and Endovascular Surgery University of California San Francisco California USA; ^16^ Department of Biomedical Engineering Carnegie Mellon University Pittsburgh Pennsylvania USA; ^17^ Department of Experimental Cardiology, Circulatory Health Laboratory Regenerative Medicine Centre, UMC Utrecht, University Utrecht Utrecht The Netherlands; ^18^ Massachusetts General Hospital Cancer Center and Harvard Medical School Boston Massachusetts USA; ^19^ Department of Biomedical Engineering, Department of Medicine Columbia University New York New York USA; ^20^ Department of Biological Sciences University of Maryland Baltimore County Baltimore Maryland USA; ^21^ Department of Pharmaceutical Sciences, College of Pharmacy and Pharmaceutical Sciences Washington State University Spokane Washington USA; ^22^ Department of Molecular and Comparative Pathobiology, Department of Neurology and Neurosurgery and The Richman Family Precision Medicine Center of Excellence in Alzheimer's Disease The Johns Hopkins University School of Medicine Baltimore Maryland USA; ^23^ Division of Cardiovascular Medicine, Department of Medicine Stanford University School of Medicine Stanford California USA; ^24^ Division of Cardiovascular Sciences, National Heart, Lung, and Blood Institute National Institutes of Health Bethesda Maryland USA; ^25^ Division of Blood Diseases and Resources, National Heart, Lung, and Blood Institute National Institutes of Health Bethesda Maryland USA; ^26^ Division of Lung Diseases, National Heart, Lung, and Blood Institute National Institutes of Health Bethesda Maryland USA; ^27^ Pittsburgh Heart, Lung and Blood Vascular Medicine Institute University of Pittsburgh School of Medicine Pittsburgh Pennsylvania USA; ^28^ Division of Cardiology and Department of Medicine University of Pittsburgh School of Medicine and University of Pittsburgh Medical Center Pittsburgh Pennsylvania USA; ^29^ Division of Pulmonary Allergy and Critical Care Medicine and Department of Medicine University of Pittsburgh Pittsburgh Pennsylvania USA

**Keywords:** Heart, lung, blood and sleep (HLBS) diseases, extracellular vesicles (EVs), therapeutics and diagnostics

## Abstract

Extracellular vesicles (EVs) carry diverse bioactive components including nucleic acids, proteins, lipids and metabolites that play versatile roles in intercellular and interorgan communication. The capability to modulate their stability, tissue‐specific targeting and cargo render EVs as promising nanotherapeutics for treating heart, lung, blood and sleep (HLBS) diseases. However, current limitations in large‐scale manufacturing of therapeutic‐grade EVs, and knowledge gaps in EV biogenesis and heterogeneity pose significant challenges in their clinical application as diagnostics or therapeutics for HLBS diseases. To address these challenges, a strategic workshop with multidisciplinary experts in EV biology and U.S. Food and Drug Administration (USFDA) officials was convened by the National Heart, Lung and Blood Institute. The presentations and discussions were focused on summarizing the current state of science and technology for engineering therapeutic EVs for HLBS diseases, identifying critical knowledge gaps and regulatory challenges and suggesting potential solutions to promulgate translation of therapeutic EVs to the clinic. Benchmarks to meet the critical quality attributes set by the USFDA for other cell‐based therapeutics were discussed. Development of novel strategies and approaches for scaling‐up EV production and the quality control/quality analysis (QC/QA) of EV‐based therapeutics were recognized as the necessary milestones for future investigations.

## 1 INTRODUCTION

**FIGURE 1:**
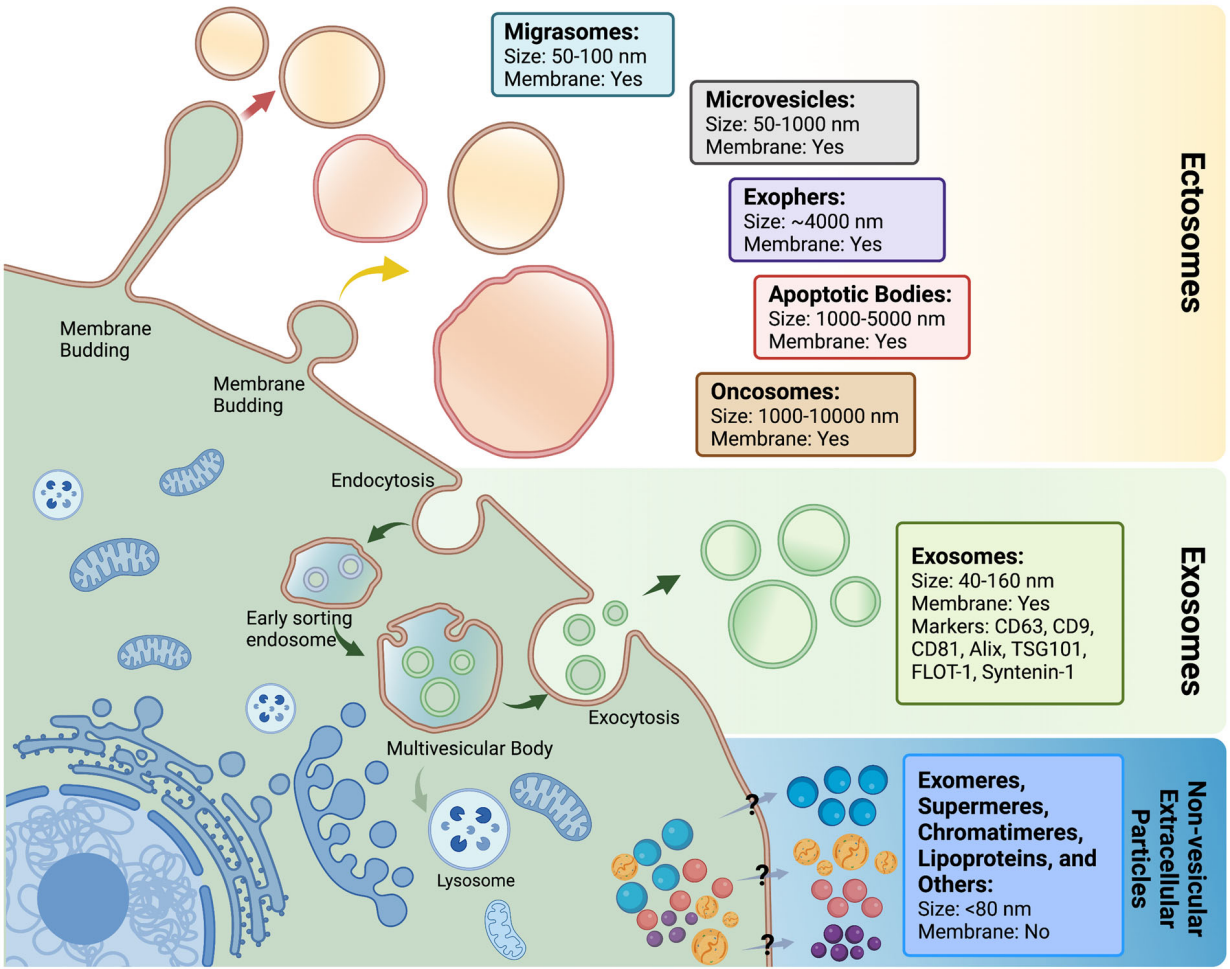
Heterogenous populations of EVs and non‐vesicular extracellular particles. Based on the biogenesis pathways, EVs can be classified into two basic categories, including exosomes and ectosomes. Exosomes are vesicles ranging ∼40–160 nm in diameter generated by the endocytic pathway and released upon fusion of endosomal multivesicular bodies (MVBs) with the plasma membrane. Ectosomes are released by the outward budding of the plasma membrane and include migrasomes, microvesicles, exophers, apoptotic bodies, large oncosomes and others, in the size range of ∼50 nm–∼5 μm. Non‐vesicular extracellular particles are non‐membranous complexes of proteins and nucleic acids with a diameter of less than 80 nm, that include exomeres, supermeres, chromatimeres, lipoproteins and several others. The mechanisms of non‐vesicular extracellular particle biogenesis are unknown, and such particles were not the focus of this workshop. All the above groups may overlap in size

Extracellular vesicles (EVs) are a heterogeneous group of nanosized lipid membrane vesicles that are released by many different cell types (Thery et al., 2018). Basically, EVs can be classified based on their biogenesis (Figure 1) into two categories, exosomes and ectosomes (Kalluri & LeBleu, 2020; van der Pol et al., 2016; Yanez‐Mo et al., 2015). Exosomes are vesicles ranging ∼40–160 nm in diameter generated by the endocytic pathway and released upon fusion of endosomal multivesicular bodies (MVBs) with the plasma membrane (Kalluri & LeBleu, 2020; van der Pol et al., 2016; Yanez‐Mo et al., 2015). Ectosomes are shed directly from the plasma membrane of diverse cell types and include microvesicles, migrasomes, exophers, apoptotic bodies and large oncosomes in the size range of ∼50 nm–∼5 μm (Buzas, 2022; van der Pol et al., 2016). Apart from removing toxic or unwanted molecular materials from cells as a means for maintaining cell homeostasis (Yanez‐Mo et al., 2015), EVs can also transfer various bioactive molecules, including proteins, nucleic acids, lipids and metabolites from donor cells to recipient cells, acting as important mediators of intercellular or inter‐organ communication at paracrine and systemic levels in both physiological and pathological conditions (Yanez‐Mo et al., 2015). Recently, many non‐vesicular (non‐EV) extracellular particles, such as lipoproteins, exomeres, supermeres, chromatimeres and several others were also found to carry distinct protein or RNA cargo and mediate intercellular communication (Mittelbrunn & Sanchez‐Madrid, 2012; Zhang et al., 2019; Zhang, Jeppensen et al., 2021).

Accumulating evidence suggests that EVs are abundantly distributed in human body fluids including blood, urine, saliva, breast milk, cerebrospinal and synovial fluid, bile and tears (Doyle & Wang, 2019). The surface and luminal content of EVs of different cellular origins are dynamically regulated by different pathophysiological states (Yanez‐Mo et al., 2015), suggestive of their potential as biomarkers for the diagnosis and prognosis of heart, lung, blood and sleep (HLBS) diseases. In addition, owing to their endogenous biogenesis, EVs are produced naturally with an extensive range of natural properties, such as high biocompatibility, limited immunogenicity, immune priming, homing, cargo diversity and capacity, enhanced stability in circulation and ability to cross blood‐tissue barriers, offering the promise that EVs may prove a unique platform for standalone therapies or as drug delivery systems (Meng et al., 2020).

Despite remarkable utility of natural EVs derived directly from stem/progenitor cells, their limitations (low yield, low purity, heterogeneous cargo) pose major hurdles in their applications for treating HLBS diseases (Vader et al., 2016). These limitations can be partially circumvented by engineering EVs with a desired therapeutic cargo, enhanced stability and efficacy, optimized tropism and precise targeting specificity to desired cells and tissues for therapeutic applications, including but not limited to vaccination and drug‐delivery (Claridge et al., 2021).

**FIGURE 2:**
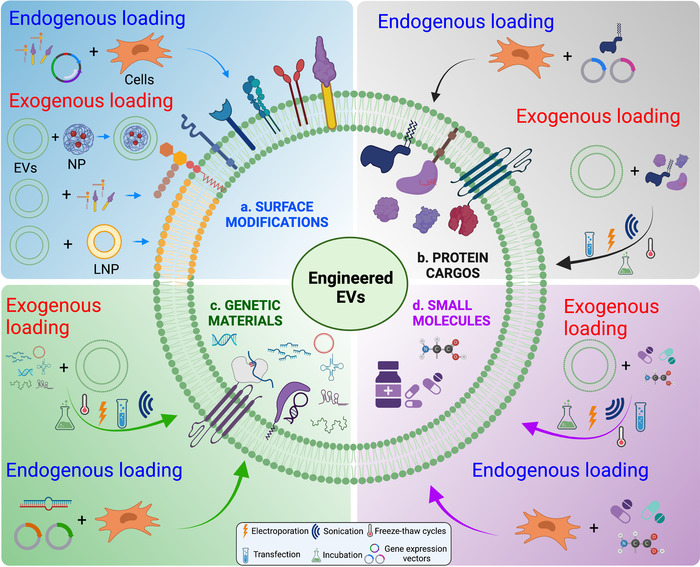
Strategies for engineering therapeutic EVs. Both EV membrane and cargo can be engineered either endogenously or exogenously for therapeutical applications. Endogenous EV engineering refers to modulating EV‐secreting (parent) cells by exposing them to stress‐induced conditions or transfecting these parent cells with exogenous compounds, such as nucleic acids, small molecules, lipids and proteins. Exogenous EV engineering is based on the modifications of isolated EVs that include exploiting the hydrophobicity of EV membranes to carry a cargo of interest on the EV surface or permeabilizing the EV membranes using approaches, such as electroporation, freeze‐thaw procedures, sonication, surfactant treatment, and chemical transfection to carry the cargo of interest as the luminal cargo in EVs. EV membranes can also be used to encapsulate cargo‐carrying nanoparticles (NPs) or EVs can be fused to cargo‐carrying lipid nanoparticles (LNPs)

Engineering of therapeutic EVs can be carried out using the following approaches (Figure 2): (1) Enrichment of endogenous molecules in EVs by culturing parent cells under specific conditions, such as hypoxia preconditioning or in particular media; (2) Gene editing of the source cells to secrete EVs carrying desired cargo; (3) Modification of EV membranes for targeted delivery to specific cells or tissues and (4) Loading isolated cell‐derived or synthetic EVs with therapeutic cargo (de Abreu et al., 2020; Piffoux et al., 2021). Importantly, engineered EVs have already been used successfully for the delivery of therapeutically relevant molecules, including miRNAs, proteins and small molecules for treating HLBS diseases in various preclinical studies (de Abreu et al., 2020).

Around ∼300 clinical trials proposing the use of EVs as diagnostics or therapeutics have been registered at clinicaltrials.gov (https://clinicaltrials.gov/) to date. Although most of these trials were based on using endogenous (unmodified) EVs, the concept of using engineered EVs in clinical trials has lately gained attention in the treatment of cancer, familial hypercholesterolemia and COVID‐19 (Kamerkar et al., 2022; Li et al., 2021; Xie et al., 2021). Despite the growing enthusiasm to explore the potential capacity of EVs as novel diagnostics and therapeutics, there are still major gaps in the knowledge and technology for engineering EVs to treat HLBS diseases.

To identify the critical knowledge gaps and research opportunities in the broad EV field and to explore the utility of native or engineered EVs for the diagnosis, prognostics and treatment of HLBS diseases, a strategic workshop was held by the National Heart, Lung and Blood Institute (NHLBI) in September 2021. A pro versus con debate on whether EVs are ready for human therapeutic applications was presented by Dr. Eduardo Marbán and Dr. Phillip Yang. The advantages of EV‐based therapeutics, including biologic plausibility, immune privilege, specific targeting and engineering versatility and the ongoing clinical trials on utilizing EVs as therapeutics or diagnostics, were highlighted by Dr. Marbán (Marban, 2018). Three major concerns were raised by Dr. Yang as challenges in the clinical application of EVs: (1) Biological unknowns including the heterogeneity of EV populations and cargo; (2) Pharmacological unknowns such as dosing, delivery route, biodistribution, pharmacokinetics and pharmacodynamics and (3) Setting‐up scalable engineering strategies and standard operating procedures for manufacturing high‐yield engineered EVs with efficient and consistent drug loading. The thus‐far‐limited regulatory guidance from the U.S. Food and Drug Administration (USFDA), which aims to ensure the safety and efficacy of cell‐derived therapeutics such as EVs, was also discussed.

To address these challenges and identify milestones for future investigations in the next decade, the presentations and discussions between experts at this workshop were focused on the following four thematic sessions: (1) engineering the membrane of EVs for HLBS disease therapy; (2) engineering the cargo of EVs for HLBS disease therapy; (3) lessons from other cell membrane‐derived vesicles and other diseases and (4) leveraging EV biology for novel diagnostics and prognostics. At the conclusion of this workshop, the group of experts (expert panel) identified key areas for future studies required to improve the engineering of EVs for HLBS diseases, emphasized the need to develop novel techniques for precision analysis of EV‐therapeutic function and identified the technological milestones necessary for scaling‐up the production of therapeutic EVs.

## 2 ENGINEERING THE MEMBRANE OF EVS FOR TREATING HLBS DISEASES

The biochemical composition of the EV membrane and the repertoire of adhesive epitopes presented by the EV membrane dictate the efficacy of EVs for use in the treatment of HBLS diseases. During this workshop, several speakers discussed the advantages and limitations of existing strategies used for designing of EV membranes and several areas with room for further improvement were identified to guide the engineering of membranes for therapeutic EVs. Deborah Nelson from the University of Chicago introduced the concept of using EVs as vehicles for functional transfer of membrane proteins to target cells, such as engineering EVs to carry the chloride‐channel Cystic Fibrosis Transmembrane Conductance Regulator (CFTR) protein as a membrane cargo to CFTR‐deficient alveolar macrophages for restoring their phagosomal microbicidal activity or transferring the functional light‐activated ORAI‐1 Ca^2+^ channels to cells lacking these channels. The presence of fluorescent cargo on EVs allows size validation and characterization of EVs using new nano‐flow cytometry‐based or high‐resolution microscopy‐based approaches. These include total internal reflection fluorescence (TIRF) microscopy or super resolution Stimulated Emission Depletion Microscopy (STED) microscopy and their roles as complementary approaches was discussed. In addition to these methodologies for characterizing EVs, newer approaches designed to address limitations of current methods for EV isolation were also discussed. Specifically, use of size‐exclusion columns (SECs), high‐pressure liquid chromatography based‐asymmetric field flow fractionation (AF4), or magnetic beads‐targeted to cargo‐protein present on EV membranes were suggested to overcome the limitations of ultracentrifugation, which is known to lead to EV aggregation and contamination by soluble proteins and may not be practical to support commercial‐scale manufacture. Several areas of improvement were identified to engineer EV‐membranes for delivery of membrane proteins to target cells, such as (1) inclusion of poly‐ethylene‐glycol (PEG) in EV suspensions to improve fusion of EVs with recipient cells, (2) using cell or virus‐derived fusion proteins to ensure EV fusion to the target‐cell plasma membrane in vivo without promoting endosomal degradation of EVs following fusion, (3) elucidating the molecular mechanism of EV generation or secretion to increase the yield of EVs carrying specific membrane‐cargo and (4) developing strategies to enrich subsets of EVs carrying therapeutically potent levels of desired protein(s) on the membrane.

In the same session, Jennifer Lang from SUNY‐Buffalo highlighted several approaches for engineering EV membranes for potential application in cardiac cell repair post myocardial infarction (MI) or chronic coronary artery disease (CAD). Based on the existing evidence, EVs‐derived from cardiac progenitor cells, such as cardiosphere‐derived cells (CDC‐EVs), were suggested to be therapeutically beneficial in reducing cardiomyocyte apoptosis, leading to reduced infarct size in preclinical models of MI (Gallet et al., 2017; Ibrahim et al., 2014; Lang et al., 2016; Maring et al., 2017). Genetic modification approaches that involve fusion of the gene sequence of a guiding protein or polypeptide, such as cardiomyocyte‐specific binding peptide (CMP) with a selected protein abundantly present on EV membranes including Lysosome‐Associated Membrane Protein 2, Isoform B (LAMP‐2b) or a tetraspanin (CD63/CD9/CD81) or glycosylphosphatidylinositol (GPI)‐anchor, were suggested to be appropriate for improving the recruitment of EVs in the myocardium. Transfection of cardiomyocytes with lentiviral vectors carrying expression plasmids for a Lamp‐2b‐CMP fusion protein was suggested to generate Lamp‐2b‐CMP expressing EVs that manifest functional properties identical to unmodified EVs; recruit more efficiently than unmodified (untargeted) EVs to murine cardiomyocytes, but not other cardiac cells; prevent cardiomyocyte apoptosis in cell culture studies in vitro and target primarily in the heart and fuse with cardiac myocytes, leading to improved cardiac function in mice in vivo (McGuire et al., 2004; Mentkowski & Lang, 2019). EVs injected intravenously into the mice are known to be rapidly cleared by CD68+ macrophages, primarily in the liver, lung and spleen. To circumvent this limitation, intra‐myocardial administration was suggested to be more efficient in promoting EV recruitment, internalization and retention in the murine myocardium. However, this route of administration is not therapeutically efficient in the clinic, suggesting the need for better approaches to engineer EV‐membranes to ensure efficient delivery of EVs administered systemically to the myocardium (Mentkowski et al., 2018).

Next, Xi Ren from Carnegie Mellon University introduced a relatively newer concept of using biomaterial immobilized‐EVs as delivery vehicles to improve therapeutic efficacy of cargo drugs. This approach relies on metabolic glycan engineering of EV surfaces to express azide‐labelled glycans using azido monosaccharide probes, such as a mannosamine analogue carrying an azide‐group (Ac_4_ManNAz), which can be included on endogenous membrane proteins of EVs as a post‐translational glycosylation modification. The azide group allowed EV detection using fluorescence imaging and efficient EV immobilization in the extracellular matrix (ECM) using copper‐free click chemistry, which is based on the reaction of a dibenzocyclooctyne moiety (DBCO) present on collagen with the azide‐group present on EV surface. Data using mesenchymal stem cell (MSC)‐generated EVs was shared to confirm that the surface azide expression on EVs (Az‐EVs) had no effect on the EV‐size distribution, endothelial uptake of EVs, or EV‐dependent endothelial cell proliferation and migration in in vitro cell culture studies. Importantly, Az‐EVs were shown to exhibit stable retention within collagen matrix expressing DBCO and be more efficient than unmodified EVs even at lower doses in promoting angiogenesis and macrophage infiltration in DBCO‐modified hydrogels subcutaneously implanted in mice (Xing et al., 2022). However, the need to modify not only the EV membranes, but also the ECM was identified as a major hurdle by the expert panel; such an approach will require more research and development before translation to the clinic. A possible systemic immune response to Az‐EVs or the modified ECM was another concern raised by the expert panel; this will also require further investigation using more comprehensive in vivo animal studies.

## 3 ENGINEERING THE CARGO OF EVS FOR TREATING HLBS DISEASES

The major limitations in the therapeutic application of bioactive molecules, including RNAs, DNAs and proteins, are associated with their poor transport across cell membrane or tissue barriers and the risk of rapid digestion by the extracellular enzymes (Murphy et al., 2019), which can be potentially circumvented through the use of an appropriate vehicle for the targeted‐delivery of these molecules to cells or organs of choice (Murphy et al., 2019). Conventional strategies to overcome these barriers involve chemically modifying these molecules to promote uptake, distribution and stability by delivering the genetic materials using engineered virus (e.g., Adeno‐Associated Virus, AAV) (Kuzmin et al., 2021) or enveloping the biological molecules within synthetic nanoparticles (e.g., lipid nanoparticles, LNPs) (Anselmo & Mitragotri, 2021). In the second session of this workshop, speakers discussed strategies for engineering nanoparticles that could potentially be used to engineer EVs as drug vehicles and highlighted the therapeutic potential of EVs with functional cargo in HLBS diseases. Lola Eniola‐Adefeso from the University of Michigan summarized three strategies for the engineering of EVs as drug delivery vehicles (Herrmann et al., 2021). The first strategy used natural EVs derived from producer cells that are manipulated to produce biological therapeutics, including RNAs, DNA, proteins, lipids and chemical drugs. Two modes of manipulating producer cells to produce EVs with desired cargo were discussed: transfection of the cells with natural or tagged genetic materials and co‐incubation of the cells with small chemical drugs. For instance, insertion of the exosome sorting miRNA motif (EXOmotif) in a miRNA of choice increases the export of this miRNA into EVs (Garcia‐Martin et al., 2022; Villarroya‐Beltri et al., 2013), while tagging the targeted proteins with a WW domain results in efficient loading of this protein into EVs (Sterzenbach et al., 2017). Incubating the cells with small molecular drugs (e.g., methotrexate) was also demonstrated to be an efficient method to package small molecular drugs into EVs (Guo et al., 2019). The major advantage of EVs derived from engineered/modified producer cells is that the native cell/tissue‐targeting properties of these EV membranes can be employed for targeted delivery of therapeutic cargo. The second strategy used post‐modified EVs that are generated by loading the isolated EVs with the cargo of choice using various strategies, ranging from passive loading (co‐incubation of EVs with desired molecular cargo) to active methods, such as thermal shock, extrusion, chemical transfection, sonication and electroporation (Witwer & Wolfram, 2021). Hydrophobic molecules, such as curcumin and doxorubicin, which can cross plasma membranes, can also infiltrate into EVs during co‐incubation under ambient conditions (Tian et al., 2014; Zhuang et al., 2011). Electroporation, which temporally induces pores in the membranes of EVs and thereby allows biological materials to enter EV lumen, has been widely used for packaging small chemical drugs (e.g., paclitaxel), RNAs, DNAs and proteins into EVs (Li et al., 2018; Zhang, Cheng et al., 2021). Although electroporation seems to be an advantageous approach for loading therapeutics into EVs, it may alter the physicochemical and morphological characteristics of EVs and induce EV aggregation. To overcome this limitation, several other strategies were proposed to maximize the loading efficiency with minimal alteration of EV characteristics. For example, co‐incubation of cholesterol‐conjugated siRNAs with EVs has been shown to load therapeutic siRNAs into EVs efficiently (Didiot et al., 2016). Sonication was also suggested to be another suitable alternative for active incorporation of RNAs, proteins and small molecules into EVs without inducing significant EV aggregation (Lamichhane et al., 2016; Rankin‐Turner et al., 2021). The third strategy used endogenous EV membranes to coat the synthetic drug carriers resulting in improved biocompatibility, prolonged half‐life in circulation and improved biological function (Fang et al., 2018; Guerrini et al., 2018; Mathieu et al., 2019). Indeed, doxorubicin‐loaded porous silicon nanoparticles coated with EV membrane have been shown to assemble inside the tumour cells by employing endogenous EV biogenesis pathways (Yong et al., 2019). In addition, synthetic nanomaterials can be coated with EV membrane in a cell‐free condition using lipid fusion or sonication (Liu et al., 2019). Also, liposomes carrying a desired drug can be fused with EVs to enhance the tissue‐specific targeting of liposomes (Sato et al., 2016). The expert panel discussed how EV membrane‐modified nanoparticles may exhibit multiple advantages over unmodified nanoparticles and EVs, including diverse and abundant drug loading, controlled release of loaded drugs and enhanced targeting specificity. Meanwhile, the need to understand the alteration of EV membrane biophysical characteristics during coating, and how these changes may interfere with the in vivo circulation of EV‐membrane coated nanoparticles were also discussed.

In the same session, James Dahlman from the Georgia Institute of Technology introduced several novel platforms using DNA‐barcoded LNPs to deliver RNA‐based therapeutics to diverse tissues. Evidence was provided to demonstrate that LNPs were comparable in size, shape and structures to prokaryotic and eukaryotic cell‐derived EVs (Witwer & Wolfram, 2021). Although targeted nanoparticles may show homing specificity to certain tissues in vitro, the same delivery specificity by nanoparticles usually is hard to recapitulate in vivo in animal models (Gregoriadis & Ryman, 1972; Paunovska et al., 2018; Zhang et al., 2018). To facilitate the discovery of specific nanoparticles targeting diseased tissues/cells in vivo, Dr. Dahlman shared his newly developed nanoparticle platform to simultaneously examine the biodistribution of many chemically distinct nanoparticles by formulating nanoparticles to carry specific DNA barcodes and sequencing these barcodes in different organs following administration (Dahlman et al., 2017). Although the platform allowed precise estimation of biodistribution in vivo, early evidence suggested insufficient functional cargo delivery due to the inefficient escape of loaded cargo from the endosome into the cytosol (1%–2%) (Gilleron et al., 2013). To circumvent this limitation, Dr. Dahlman shared another system, named Fast Identification of Nanoparticle Delivery (FIND), which was created by co‐formulating different LNPs with unique DNA barcodes and functional RNAs, such as siRNAs, guide RNAs or mRNAs of Cre or Cas9 (Sago et al., 2018). The screening of hundreds of chemically distinct LNPs using the FIND system led to identification of several LNPs that specifically deliver functional cargo into splenic endothelial cells (Sago et al., 2018), hepatic endothelial cells (Paunovska et al., 2019), Kupffer cells (Paunovska et al., 2019) and lung endothelial cells (unpublished) following systemic administration. A similar strategy using DNA‐barcoded LNPs carrying an aVHH mRNA in various humanised or primatized mice was shown to result in the species‐dependent uptake and processing of different LNPs (Hatit et al., 2022). Using another cluster‐based LNP screening system, the efficient nebulization‐based delivery of therapeutic RNAs to lung was found to need distinct LNP formulations compared to the ones used for systemic delivery (Lokugamage et al., 2021), suggesting that several strategies for designing LNPs would be needed for engineering therapeutic cargo‐loaded synthetic EVs. The expert panel also discussed whether it would be possible in future to barcode EV populations derived from different cell types or biogenesis pathways to screen the target cells of these EV populations in vivo.

Next, Tianji Chen from the University of Illinois at Chicago shared the concept of using endogenously engineered endothelium‐derived EVs to treat pulmonary hypertension (PH) in rodent models. Pulmonary vascular endothelial cells (PVECs) and pulmonary artery smooth muscle cells (PASMCs) are two cell types in the lung that are critical to maintaining vascular homeostasis; derangement of their intercellular communication is a key step in the pathogenesis of pulmonary vascular structural remodelling in all forms of PH (Gao, Chen et al., 2016). Importantly, EVs released from PVECs under hypoxic conditions have been shown to have higher expression of miR‐210‐3p (a well‐known pro‐proliferative miRNA), and miR‐212‐5p (a recently identified anti‐proliferative miRNA) and to induce PASMC proliferation, pulmonary vascular remodelling and PH in mice (Chen et al., 2022b). Dr. Chen shared published and unpublished findings to show how endogenous cell‐derived EVs carrying either miR‐212‐5p mimetics or miR‐210‐3p antagonists can be engineered to test their potency in treating PH in rodent models (Chen et al., 2022a). She demonstrated how this can be achieved using a lentiviral system to express miR‐212‐5p or antagonists against miR‐210‐3p with an EV‐sorting sequence (GGAG) at the 3′‐end to direct them into endogenous EVs prior to their release from PVECs. Finally, Dr. Chen showed that intratracheal instillation of these engineered PVEC‐EVs into PH rodents led to efficient delivery of miR‐212‐5p and/or miR‐210‐3p antagonists into the lung and lung vessels, leading to attenuation of PH. These results highlighted the therapeutic potential of engineered PVEC‐EVs in the treatment of PH and the need for development of improved strategies to engineer such EVs for other HLBS diseases.

Nykia D. Walker from the University of Maryland Baltimore County discussed the potential of MSC‐based therapies, including administration of MSC‐derived EVs or direct transfusion of MSCs to deliver EVs to the target cells in vivo to promote tissue repair (Munoz et al., 2013). MSCs, a population of cells identified based on a distinct repertoire of membrane proteins expression reside in various organs and can be derived from multiple tissues/cells (Dominici et al., 2006; Pittenger et al., 2019, 1999). MSCs can migrate to injured sites, differentiate into various functional somatic cells, believed to promote tissue repair and manifest potent immunomodulatory properties through EV‐mediated remote intercellular communication (Borger et al., 2017; Kean et al., 2013; Song et al., 2020). Dr. Walker showed that TLR4 priming of MSCs by Lipopolysaccharide (LPS) treatment induced a pro‐inflammatory phenotype leading to secretion of IL‐6, IL‐8 and EVs enriched with miR‐146a, while TLR3 priming led to anti‐inflammatory MSCs producing nitric oxide (NO), indoleamine 2,3‐dioxygenase (IDO), prostaglandin E2 (PGE‐2) and EVs abundant in miR‐221‐3p (Borger et al., 2017; Levy et al., 2020; Walker et al., 2019). Additionally, Dr. Walker identified a subset of patients with progressively elevated MSC migration in peripheral blood at various stages of orthotopic liver transplantation surgery and recovery (Walker et al., 2017). This finding supports the concept that MSCs are both capable of retrieving inflammatory signals and recruiting to injured tissues. The anti‐inflammatory effects and the ability of differentiation to somatic cells (e.g., cardiomyocytes) suggest that MSCs can be useful in therapies for HLBS diseases (Diederichsen, 2017; Guo et al., 2020). Dr. Walker discussed an emerging approach, which uses MSC‐derived EVs as cell‐free regenerative therapeutics against HLBS diseases by delivering functional cargo (e.g., miRNAs) with versatile protective effects including anti‐inflammation, anti‐apoptosis, anti‐fibrosis and pro‐angiogenesis to diseased cells (Panda et al., 2021; Sun et al., 2021). Dr. Walker also shared some new evidence highlighting how local MSC‐EV delivery can be achieved in future by direct transplantation at the injured site or intravenous administration of engineered MSCs with pertinent EV cargo to promote tissue repair. This strategy of directly engineered MSCs transplantation may sound more therapeutically potent than MSC‐EVs administration, however, such an approach can be limited by the replicative senescence and aging of MSCs, and therefore, future in vivo studies are needed to further explore the therapeutic potential of directly engineered MSC‐based therapy in HLBS diseases.

## 4 LESSONS FROM OTHER CELL MEMBRANE‐DERIVED VESICLES AND OTHER DISEASES

Studies done over the last decade have led to better understanding of the mechanisms underlying EV biogenesis, the diversity and sorting of cargo proteins/DNA/RNA in EVs and the biological function of endogenous EVs released by different types of cells during both disease and healthy conditions in vivo (Aatonen et al., 2012; Andaloussi et al., 2013; Catalano & O'Driscoll, 2020; Kalluri & LeBleu, 2020; Lawson et al., 2016; Mathieu et al., 2019; Raposo & Stoorvogel, 2013; Shah et al., 2018; Sung et al., 2020; van der Pol et al., 2016; van Niel et al., 2018; Xunian & Kalluri, 2020). During this workshop, a session was focused on identifying how EV‐biology in other diseases can be harnessed to develop EV diagnostics for HLBS diseases or engineer EVs that are more efficient for therapeutic use in HLBS diseases. Peter Kurre from Children's Hospital of Philadelphia shared the current understanding of the role that EVs play in regulating hematopoietic function in the bone marrow niches, and how this is altered in the progression of acute myeloid leukaemia (Akinduro et al., 2018; Boyd et al., 2017; Doron et al., 2018; Miraki‐Moud et al., 2013; Schepers et al., 2013). Findings suggest that EVs released in the bone marrow contribute to the crosstalk between hematopoietic stem cells (HSCs) and acute myeloid leukaemia (AML) cells, and this crosstalk can be used as a model to understand the role of EVs in regulating the microenvironment within the bone marrow niches (Abdelhamed et al., 2019, 2021; Doron et al., 2019; Hornick et al., 2016, 2015; Huan et al., 2015, 2013; Viola et al., 2016). Studies using an AML xenograft model in mice have identified that EVs from human AML cells transport human transcripts such as FLT3 and CXCR4 to murine hematopoietic stem progenitor cells (HSPCs) in the bone marrow and how AML EVs carrying miRNAs regulate function of residual HSPCs by targeting the critical transcription factor c‐Myb, leading to progression of AML (Abdelhamed et al., 2021; Hornick et al., 2016). These studies also identified that EVs derived from AML cells‐carry a different set of miRNAs which regulate the function of HSCs by targeting the mTOR‐pathway, leading to the arrest of protein synthesis (Abdelhamed et al., 2019, 2021). Interestingly, in addition to modulating the bone marrow function in other types of blood cancers, EVs have also been shown to play a role in regulating HSPC function under healthy homeostatic conditions (Aliotta et al., 2012; Boysen et al., 2017; Corrado et al., 2014; Goloviznina et al., 2016; Huang et al., 2021; Kumar et al., 2018; Roccaro et al., 2013; Szczepanski et al., 2009; Wen et al., 2016). Evidence provided by Dr. Kurre suggested that much can be learned by understanding the role of HSPC‐derived EVs in affecting the fate of other HSPCs by promoting differentiation to various cell types, self‐renewal or senescence, endothelial activation and angiogenesis (Hurwitz et al., 2020). For example, HSPC‐derived EVs carrying the stem cell marker CD133 primarily target stromal cells, but not other HSPCs (Bauer et al., 2011). Also, disruption of EV biogenesis leads to impaired quiescence, self‐renewal, stress resistance and increased apoptosis in HSPCs (Alexander et al., 2017; Gu et al., 2016; Hu et al., 2022), most likely due to the impairment of autocrine signalling mediated by EV‐associated stemness factors thrombopoietin (TPO), angeopoeitin‐like2 (Angptl2) and possibly ligands for other receptors present on HSPCs (Hurwitz et al., 2020; Teng & Fussenegger, 2020). Emerging evidence was also shared to support a role for EVs in paracrine signalling between HSPCs, megakaryocytes and neutrophils (Hurwitz et al., 2020). Similarly, CD34+ EVs released by CD34+ HSPCs are known to be enriched specifically in miRNAs and certain membrane integrins, suggesting that CD34+ EVs can be used for delivery of miRNAs to target cells based on the integrin expression on the EV membrane (French et al., 2017; Sahoo et al., 2011). The expert panel realized that better understanding of these mechanisms regulating EV‐dependent autocrine and paracrine signalling in HSPCs could possibly be harnessed for designing EVs that are more efficient for tissue‐specific targeting in HLBS‐diseases therapy (Qin & Dallas, 2019).

Zhenjia Wang from Washington State University shared the concept of using neutrophil membrane to create nanovesicles for treating lung inflammation in the setting of acute lung injury (ALI). This strategy involves neutrophil disruption by nitrogen cavitation followed by separation of membrane components using series of ultracentrifugation steps and extrusion through a membrane of specific pore size (∼50–200 nm) to generate neutrophil‐membrane‐derived nanovesicles (Gao, Chu et al., 2016). These neutrophil‐derived nanovesicles express major adhesion molecules and receptors on the neutrophil membrane, such as CD18 (β2‐integrin chain), toll like receptor‐4 (TLR4), P‐selectin‐glycoprotein‐ligand‐1 (PSGL‐1) and CD49d (α4‐integrin chain). Akin to the parent neutrophils, these nanovesicles were shown to recruit only to inflamed cremaster muscle vasculature in TNF‐treated mice and lungs of intra‐tracheal LPS‐treated mice, but not to healthy tissue in vivo (Gao, Chu et al., 2016; Gao, Wang et al., 2020; Wang, 2016; Wang et al., 2014), suggesting their potential for delivering therapeutics specifically to the site of inflammation. Indeed, recent evidence shared by Dr. Wang suggested that neutrophil‐derived nanovesicles carrying pro‐resolving mediators Resolvin D1 (RvD1) or D2 (RvD2) as membrane cargo, reduced the time to resolution of lung injury, cytokine storm in the lung and endothelial ICAM‐1 expression and also promoted neutrophil apoptosis in murine models of ALI (Gao, Wang et al., 2020). Similarly, neutrophil membrane‐derived nanovesicles carrying RvD1 on the membrane and antibiotic (ceftazidime) as luminal cargo were shown to significantly reduce the bacterial load, neutrophil infiltration, cytokine storm and injury in the lungs of mice following bacterial infection (Gao, Wang et al., 2020). Some of the concerns raised by the expert panel centred around the lack of specificity of these nanovesicles to inflamed endothelium in the lung versus other vascular beds, ability to scale‐up the method of nitrogen cavitation, composition of nanovesicle formulation (exosomes, microvesicles or a mixture of both), the possibility of these nanovesicles promoting immune response and the fate of these nanovesicles following adhesion to endothelium. Therefore, several limitations and gaps in knowledge of their mechanism of action need to be addressed before these nanovesicles can be used for treatment of lung and other HLBS diseases (Gao, Dong et al., 2020; Gao et al., 2017).

Ionita Ghiran from Beth Israel Deaconess Medical Centre discussed the biology underlying generation of red blood cell (RBC)‐derived EVs, how they contribute to cell‐cell communication and how they can potentially be used in HLBS diseases therapy. Besides electron microscopy, newer techniques like nanoflow cytometry, super‐resolution fluorescence microscopy, dark‐field microscopy and magnetic levitation relying on presence of specific antigens on EVs were described as major approaches available for RBC‐derived EV analysis or identification of EV‐cargo, such as miRNAs in both high and low resource settings (Babatunde et al., 2018; Oliveira et al., 2020). RBCs are the most abundant cell type in the blood (∼5 × 10^6^/μl of blood), carry a negatively charged membrane (enriched with sialic acid, glycophorin‐A (GPA) and glycocalyx), lack organelles and contain a limited number of signalling proteins and small RNAs, such as miR‐451 in the cytosol (Kuo et al., 2017). RBC‐derived EVs are among the most abundant species of circulating EVs in the blood (Donadee et al., 2011). Although RBCs lack the signalling mechanism required for endocytosis or exocytosis, circadian‐driven complement (C5b‐9)‐mediated ectocytosis is believed to be the main mechanism for daily generation of RBC‐derived EVs in the blood circulation both under healthy and inflammatory states (Donadee et al., 2011; Iida et al., 1991; Karasu et al., 2018; Thangaraju et al., 2021). In collaboration with Dr. Das, the targets of circulating RBC‐EVs were recently identified using a cre‐loxp murine model (Valkov et al., 2021). RBCs isolated from an erythroid‐specific‐cre (EpoR‐cre) mice underwent complement activation, and the resulting EVs were purified and intravenously administered into Rosa26‐mTmG reporter mice. CRE‐containing RBC‐EVs that are taken up by the recipient cells and bypass the lysosomal compartments, will promote a tomato‐to‐green fluorescence conversion of the recipient cells. The results showed that physiologically, RBC‐EVs fuse to megakaryocyte‐erythroid progenitor cells in the bone marrow, pericytes in the blood vessels, kidney cells, cardiomyocytes and during inflammatory condition, microglia in the brain. Based on these findings, several questions were raised by the expert panel. How can RBCs be loaded with a therapeutic cargo to generate cargo‐carrying RBC‐EVs efficiently? How do the RBC‐derived EVs fuse to the target cell membrane? How do they deliver the cargo into the cytosol by bypassing the lysosomal system? Can RBC‐derived EVs promote an immune response (Camus et al., 2015; Donadee et al., 2011; Hierso et al., 2017; Olatunya et al., 2019)? The better understanding of all these endogenous mechanisms could be harnessed to use RBC‐derived EVs for therapeutic delivery in treatment of HLBS‐diseases.

Shannon Stott from the Harvard Medical School was the last speaker in this session and introduced the concept of using microfluidic platforms to isolate rare populations of cell‐specific EVs from the blood of cancer and COVID‐19 patients (Li et al., 2015; Reategui et al., 2015, 2018). Dr. Stott discussed EV isolation from small sample volumes (a few hundred microliters) using an affinity‐based capture methodology wherein interactions between EVs and capture moieties are enhanced by microfluidic manipulation. Specifically, a microfluidic device (^EV^HB‐Chip) was presented with arrays of tortuous channels functionalized with antibodies targeted against epitopes (such as PODO, EGFRvIII or PDGFR) expressed on EVs of interest (Reategui et al., 2018). Such a microfluidic system functionalized with a thermo‐responsive biomaterial presenting antibodies attached via a linker was shown to capture 80% of EVs present in complex biofluids, such as plasma suspensions (Reategui et al., 2018). This approach was suggested to be significantly more efficient than the traditional methods of Ab‐bound magnetic beads or ultracentrifugation of serum (Reategui et al., 2015), and proposed to be very efficient in capturing (isolating) rare populations of tumour cell‐derived EVs—as few as 100 EVs/100 μl of sample (Reategui et al., 2018). Several examples were shown to demonstrate how microfluidic‐based EV isolation could be useful in RNA‐seq based biomarker or diagnostic studies in brain glioblastoma (brain tumour) patients. RNAs for major genes, such as EGFRvIII, were shown to be significantly enriched in EVs isolated using ^EV^HB‐Chip compared with ultracentrifugation (Reategui et al., 2018). Evidence showed that RNAseq analysis of EVs isolated using ^EV^HB‐Chip functionalized with an antibody cocktail against EGFRvIII, Cetuximab, PDPN and PDGFR led to the identification of at least 100 differentially expressed genes in EVs isolated from glioblastoma patients compared with healthy controls, and allowed discrimination between glioblastoma patients with progressive versus pseudoprogressive disease states based on EV‐RNA signatures. Additionally, the small volume of sample (100 μl of plasma) needed for microfluidic‐based EV isolation allows this approach to be used for biomarker discovery in small volumes of plasma from paediatric medulloblastoma patients. Preliminary evidence was also provided to show how ^EV^HB‐Chip can be functionalized with ACE2 receptor for SARS‐Cov2 virus or cell‐specific Ab cocktail (CD3, CD4 or CD8 T‐cells) to capture virus or cell‐specific EVs from the plasma of COVID‐19 patients, and how these EVs can be used for RNAseq to potentially identify patients suitable for immunotherapy or diagnosis of COVID‐19 based on detection of viral RNA in EVs or plasma. Although the microfluidic systems seem to allow high purity isolation of EVs from small volume plasma samples, several areas for further research and development were identified by the expert panel, such as the need to combine with sophisticated hardware and software, select optimum Ab cocktails, address limitations associated with the usage of whole blood and investigate how haemolysis, activation of blood cells, coagulation during blood or plasma storage may affect the efficiency of EV isolation (Tessier et al., 2021; Wong et al., 2017).

## 5 LEVERAGING EV BIOLOGY FOR NOVEL DIAGNOSTICS AND THERAPEUTICS

EVs are abundant in all body fluids (Yanez‐Mo et al., 2015), offering promise as potential biomarkers for novel diagnostics and prognostics (Yuana et al., 2013). The last session of this workshop was focused on the potential of EVs to serve as biomarkers and therapeutics for HLBS diseases.

Gordana Vunjak‐Novakovic from Columbia University introduced the concept of using integrated platforms with micro‐sized human tissues linked by vascular perfusion to study the crosstalk by EVs between different tissue/organ systems (Ronaldson‐Bouchard et al., 2022). She reported the modular ‘multiorgan on a chip’ (MOC) platform configured with human tissues that were derived from induced pluripotent stem cells (iPSC): bone, innervated skin, heart and liver. For MOC to be a useful platform for biomarker and therapeutic studies, iPSC‐derived human tissues need to be sufficiently matured to achieve functional resemblance to their native counterparts. To meet this requirement, each tissue was grown and maintained in its optimal environment (medium composition, molecular and physical regulatory signals) and matured to an adult‐like phenotype over approximately 4 weeks of culture. For example, the maturation of heart muscle was achieved by subjecting the forming tissue to electrical stimulation of an increasing intensity. This protocol resulted in human heart tissue displaying several adult CM‐like signatures including gene expression, ultrastructure, metabolism and calcium handling (Ronaldson‐Bouchard et al., 2018). Another example is the maturation of iPSC‐derived skin tissue that was achieved by cultivation on air‐liquid interface. The matured tissues were then linked by vascular flow containing circulating immune cells, cytokines and EVs, with an endothelial barrier separating the intratissue and intravascular compartments. This way, each tissue maintained their phenotypes over long culture times, while communicating with other tissues across endothelial barriers and vascular circulation. This biomimetic system recapitulated many aspects of interorgan communication in the human body (Ronaldson‐Bouchard et al., 2022). To document crosstalk between tissues by EVs, one of the tissues (heart) was generated from hiPSCs transfected with a green fluorescent protein (GFP)‐labelled CD63 EV reporter, enabling the tracking of a non‐ubiquitous organ‐specific marker of known origin. CD63‐EVs secreted by heart tissues were found in all tissues after 2 weeks of culture in the MOC, suggestive of EV‐mediated inter‐organ crosstalk. Similarly, immunofluorescence imaging of the vascular barrier beneath the heart tissue after 2 weeks showed EV uptake by endothelial cells (Ronaldson‐Bouchard et al., 2022). Dr. Vunjak‐Novakovic also highlighted the therapeutic effects of EVs derived from iPSC‐CMs on the regeneration of injured heart muscle after MI (Liu et al., 2018). These iPSC‐CM‐derived EVs were found to be enriched with cardiac‐ and vascular‐specific miRNAs that modulate the cardiac response to injury. Numerous other studies support the therapeutic use of miRNAs to control gene expression through specific targeting of mRNAs (Liu et al., 2021). Based on these studies, the current challenges and considerations relevant for translating the use of EVs as diagnostics or therapeutics were discussed, including the use of physiologically relevant in vitro models (e.g., MOC) and improved understanding of the complexity of the EV biology.

Next, Robert L. Raffai from the University of California at San Francisco discussed the biological roles of macrophage‐derived EVs and their potential applications as biomarkers and therapeutics in cardiometabolic diseases, such as atherosclerosis. Macrophages are primary innate immune cells that reside in nearly all tissues with tissue‐specific characteristics (Gentek et al., 2014). Macrophage‐derived EVs can deliver proteins, lipids and genetic materials to recipient cells, which play pivotal roles in the processes of vascular inflammation (Nguyen et al., 2018). However, the contents and functions of macrophage‐derived EVs are likely to be as diverse as the phenotype of their parental macrophage subtypes, as well as their inflammatory state. Dr. Raffai shared his recent findings demonstrating that macrophages cultured in hyperglycaemic conditions produce EVs that can contribute to accelerated atherosclerosis resembling what has been reported in diabetes (Bouchareychas et al., 2021; Nagareddy et al., 2013). EVs derived from the bone marrow‐derived macrophages (BMDM) exposed to high glucose media communicated glycolytic metabolism and inflammatory signalling to naive BMDMs, driving M1 polarization (Bouchareychas et al., 2021). Additionally, adoptively transferred macrophages‐derived EVs were shown to be taken up by recipient cells in the bone marrow and aorta, but the main retention was primarily in the liver and spleen. Notably, intraperitoneal injections of macrophage‐derived EVs isolated from hyperglycaemic, but not control media, induced significant expansion of the common myeloid progenitor and granulocyte‐macrophage progenitor cells in both the bone marrow and spleen of ApoE^−/−^ mice, which further augmented leukocyte counts in the circulation and contributed to accelerate both spontaneous and diet‐induced atherosclerosis (Bouchareychas et al., 2020). EVs derived from high glucose‐exposed macrophages or diabetic patients’ plasma were enriched in miRNA‐486‐5p, which regulates haematopoiesis by targeting ABCA‐1 expression (Bouchareychas et al., 2020). In contrast, macrophages stimulated with IL‐4 secrete EVs enriched with a cluster of anti‐inflammatory miRNA‐99a/146b/378a that foster M2 polarization in recipient macrophages by augmenting mitochondrial metabolism and oxidative phosphorylation. Infusions of such M2‐exosomes into ApoE‐/‐ mice reduced western diet‐induced haematopoiesis in the bone marrow and the spleen, as well as inflammatory activity in monocytes and macrophages that led to the resolution of atherosclerosis (Bouchareychas et al., 2020). The expert panel identified the need for further studies to explore how the anti‐inflammatory effects of M2‐macrophage‐derived EVs can be harnessed for therapy in cardiometabolic inflammation and how the miRNA cargo in macrophage‐derived EVs can serve as reliable biomarkers for HLBS diseases.

Next, Pilar Martin from the Spanish National Centre for Cardiovascular Research accentuated a circulating EV‐borne miRNA as a novel biomarker for the detection of acute myocarditis, which is an inflammation of the myocardium triggered by multiple causes, including infectious pathogens or autoimmune disorders, and may develop into the dilated cardiomyopathy (DCM), or cause sudden cardiac death (Cooper, 2009; Felker et al., 2000; Gannon et al., 2019). Previous studies have suggested that the type 17 helper T (Th17) lymphocyte response may be a prominent immunophenotype in human myocarditis and the sequela DCM (Myers et al., 2016). MiRNAs have emerged as epigenetic regulators and novel biomarkers for cardiac inflammation in myocarditis and other cardiovascular diseases (Boon & Dimmeler, 2015; Heymans et al., 2016). Evidence demonstrated that Th17 cells were induced in experimental autoimmune myocarditis (EAM) mice by the specific expression of mmu‐miR‐721, which directly targets Peroxisome proliferator‐activated receptor gamma (PPARγ) mRNAs and enhances RAR‐related orphan receptor gamma T (RORγt) expression and IL‐17 secretion in CD4^+^ T cells (Blanco‐Dominguez et al., 2021). Strikingly, mmu‐miR‐721 expression was shown to be specifically elevated in the plasma of mouse EAM and Coxsackievirus (CVB3)‐induced myocarditis models. The mmu‐miR‐721 was preferentially exported into EVs by Th17 cells in the circulation of EAM mice and silencing of mmu‐miR‐721 by miRNA sponge vectors inhibited RORγt expression and Th17 immune response, which eventually attenuated EAM development in mice (unpublished data). More importantly, hsa‐miR‐Chr8:96 (human homologue of mmu‐miR‐721) was also increased in the plasma of myocarditis patients as compared to acute myocardial infarction (AMI) patients or healthy controls, thus suggesting that the plasma or EV‐associated hsa‐miR‐Chr8:96 can be a reliable biomarker to distinguish acute myocarditis from MI patients or healthy controls. During the panel discussion, Dr. Martin also highlighted preliminary data in which plasma hsa‐miR‐Chr8:96 expression has a significant correlation with COVID‐19 mRNA vaccine‐induced but not COVID‐19‐induced myocarditis in two small cohorts of vaccinated individuals and SARS‐Cov2 patients, respectively (unpublished data), suggestive of a possibly different pathological mechanism enabling COVID‐19‐induced myocarditis.

The last speaker in this session, Joost P. G. Sluijter from the University Medical Centre Utrecht Regenerative Medicine Centre, highlighted the therapeutic roles of EVs in cardiac tissue repair. Dr. Sluijter shared published and unpublished findings to demonstrate how EVs are believed to contribute to the beneficial effects of stem cell therapy. The percutaneous intracoronary delivery of EVs was shown to protect the myocardium against ischemia‐reperfusion‐induced injury in both mouse and canine models (Wang et al., 2021). Also, the direct injection of cardiac progenitor cell (CPC)‐derived EVs into the myocardium was shown to manifest cardioprotective roles against MI‐induced cardiac injury by stimulating cardiovascular cell proliferation (Maring et al., 2019). Several examples were highlighted by Dr. Sluijter to justify the need for our better understanding of the biological processes responsible for the therapeutic function of EVs and the EV cargo (Roefs et al., 2020; Yang et al., 2019). The extracellular matrix metalloproteinase inducer (EMMPRIN) was shown to be required for the angiogenic effects of CPC‐derived EVs during tissue repair (Vrijsen et al., 2016). EVs isolated using SEC were shown to manifest more intact biophysical properties and higher functionality compared to EVs isolated by ultracentrifugation (Mol et al., 2017). Calcium ionophore‐stimulated EVs from CPCs were shown to be less efficient in activating AKT and ERK signalling pathways in endothelial cells compared to EVs generated without calcium stimulation (Hessvik & Llorente, 2018). Unstimulated EV‐specific exosomal protein, pappalysin‐1 (PAPP‐A), was suggested to be indispensable for the cardioprotective effects of CPC‐derived EVs (Barile et al., 2018). Dr. Sluijter also introduced three different EV engineering strategies. The first strategy relied on inducible loading of EV cargo by expressing the desired cargo (e.g., Cre) fused with DmrC and cell membrane‐associated protein fused with DmrA in the EV‐generating parent cells. Following treatment of parent cells with a ligand, DmrC and DmrA form a heterodimer, which eventually results in the packaging of desired cargo in the EVs. The second strategy was based on tagging the therapeutic EV membrane with a cardiac homing peptide (similar to the strategy proposed by Jennifer Lang), which significantly increased the retention of modified EVs within the heart muscle (Vandergriff et al., 2018; Wang et al., 2021). The third strategy (similar to the strategy proposed by Zhenjia Wang) relied on cell‐derived nanovesicles fabricated from cell bodies using sonication, serial extrusion and SEC isolation as a functional EV alternative (Ilahibaks et al., 2019).

## 6 FUTURE CHALLENGES AND CONCLUDING REMARKS

The knowledge shared by the speakers and questions raised by the expert panel led to the identification of current knowledge gaps and challenges, as well as future opportunities and potential milestones to guide investigations for improving the engineering of the therapeutic EVs for HLBS‐diseases (Table 1). EVs are currently being tested for their potential clinical application based on their biological origin, lower immunogenicity, versatility in engineering the membrane or cargo and their potential for tissue‐specific targeting (Anselmo & Mitragotri, 2021; Herrmann et al., 2021; Kalluri & LeBleu, 2020; Mentkowski et al., 2018; Murphy et al., 2019). However, limited knowledge of the heterogeneity of EV populations or their corresponding cargos, challenges in minimizing off‐target effects and improving tissue‐specific targeting, short half‐life and bioactivity in the circulation, selection of the dose (EVs vs. therapeutic cargo), dosage strategy (size of dose vs. frequency), route of administration (intravenous or intratracheal or intramyocardial), poorly understood pharmacokinetics or pharmacodynamics in vivo, unknowns related to the scale‐up of manufacturing for the pharmaceutical grade EVs, challenges in ensuring batch to batch reproducibility and loss of EV function during cryopreservation (Tessier et al., 2021) pose challenges in the translation of EVs for the therapy of HLBS diseases. Besides functional optimization of engineered EVs, the process would also need to match the milestones for cell‐derived products set by regulatory agencies, such as ensuring control, standardization and reproducibility of EV sources (parent cells used for EV production), and the methods used for EV production, including appropriate product test methods, to ensure the reproducibility of the therapeutic effects of engineered EVs. It is important to realize that methods of EV preparation suitable for pilot studies (e.g., making use of ultracentrifugation) may not be practical for commercial scale manufacture. It is also crucial to understand that properties of EVs obtained by pilot scale methods may not be at all comparable to larger scale methods based on an alternative technology. Many of the same issues encountered with somatic cell therapies also apply to EVs. These include their inherent risk (in that terminal sterilization procedures such as gamma irradiation or autoclaving cannot be applied), short product half‐life, analytic complexity of the therapeutic and unknown critical quality attributes (CQAs) perhaps including EV size distribution (which may influence their pharmacologic disposition and thus optimal dosing regimens), composition and defining complete elements of the EV cargo prior to translation of engineered EVs to the clinic. It is likely that the complete set of CQAs still remains to be determined and therefore, further work in this area is essential. To mitigate all these limitations, the expert‐panel proposed several strategies: (1) synergy between investigators to standardize the platforms for the EV‐generation, which can be partially achieved by establishing immortalized cell lines for EV‐generation analogous to the monoclonal antibody generation; (2) advancing analytical techniques analogous to single cell‐RNA or genomic sequencing to characterize engineered EVs at single EV‐level and (3) developing an ATLAS of cells capable of generating EVs and types of cells/tissue targeted by these EVs in vivo. Several suggestions were made to address the challenges associated with cryopreservation of plasma or EVs, such as administering autologous primary cells loaded with therapeutic cargo that may eventually generate EVs in vivo. However, this strategy was identified to be based on a non‐trivial precision medicine approach and more elaborate studies would be needed to identify long‐term deleterious effects arising from administrating engineered autologous cells. Alternatively, EVs, such as neutrophil membrane‐derived nanovesicles, were suggested to be made on demand to avoid long term storage; however, much remains to be learned about the efficacy, mechanism of action, target‐cell specificity and half‐life of such synthetic EVs.

**TABLE 1:** Challenges and future research opportunities for engineering EV‐based therapeutics for HLBS diseases

Category	Challenges	Future Research Opportunities
Basic EV biology	Low yield of EVs from producer cells	Increasing EV secretion from producer cells
		Elucidating EV biogenesis and uptake mechanisms
	Unspecified EV targeting properties	Identifying homing/targeting specificities of EVs with different origins or conditions
	Heterogeneity of EV subpopulations and their cargo	Elucidating precise mechanisms governing the sorting and integration of biological materials into EVs
		Identification and enrichment of different EV subpopulations
	Unknown active biomolecules of natural EVs	Identifying active ingredients of natural EVs with therapeutical benefits
	Novel EV alternatives, such as nanovesicles, exomeres and supermeres	Investigating biogenesis and uptake of these EV alternatives
EV engineering	Consistency and efficiency of cargo loading	Efficient cargo loading/release from producer cells
		Rigorous methodologies for exogenous cargo loading to EVs
		Specific enrichment of EVs carrying potent therapeutic cargo
	Contamination of other EV subpopulations	Identifying the specific properties of different EV subpopulations and optimizing the EV isolation protocol
	Side‐effects of other biomolecules in EVs	Minimizing the ratio of other biomolecules in EVs
	Low efficiency of functional EV delivery	Efficient uptake/fusion with target cells
		Escaping the endosomal degradation
	Unspecific targeting	Specific targeting with minimized off‐target effects
	Short half‐life of transfused EVs	Transfusion of donor cells producing therapeutical EVs
	Novel EV alternatives, such as nanovesicles, exomeres and supermeres	Exploring the engineering ability of these EV alternatives
Preclinical and clinical studies	Safety and efficacy of EV administration	Dosage strategy (size of dose and frequency) for each delivery/targeting
		Route of administration
		Biodistribution
		Pharmacokinetics and pharmacodynamics
	Systematical characterization of EV therapeutics	Precision methods for the analyses of EV composition and function
		Single‐EV level analysis
	Differences between small animal models and human patients	Large animal models (non‐human primates)
		Physiological in vitro human models for studying EV biology in human
EV manufacturing	Scalability and reproducibility of EV manufacturing	Scalable engineering strategy
		Scalable EV production procedure
		Scalable EV isolation protocol
		Reproducibility of EV manufacturing
	Storage conditions preserving EV function	Standardized procedure for EV storage and shipping
	Official standards for EV‐based therapeutics	Standardizing critical quality attributes of EVs
		Common regulatory guidance that ensures the safety and efficacy of EV administration

In this workshop, engineering the EV‐membrane or the cargo were presented as potential novel approaches that hold promise for engineering personalized or disease‐specific EVs for HLBS disease therapy. The potential EV diversity in selection of membrane or cytosolic cargo could be applied to various HLBS disorders. However, gaps in knowledge of the both the biological function and the approaches used for engineering or analysis of such EVs need to be resolved before such engineered EVs can translate to the clinic. Specifically, the development of necessary technologies would be required for precision analysis of key aspects such as EV content, fusion and uptake of engineered EVs by the target tissue, delivery of therapeutic cargo to the target‐tissue and the effect on the biological function of the target tissue or cell. Also, an understanding of the effect of engineered EV‐membrane or the cargo on the biophysical, functional and pharmacologic properties of EVs would be essential. Especially, how altering the EV membrane composition affects the EV lipid composition, and whether it affects EV functionality or uptake remains to be determined. Biological EVs with engineered membrane or cargo may also contain several other endogenous proteins or nucleic acids; therefore, the potential side effects of endogenous biomolecules in engineered EVs would also need to be minimized. Several key aspects of engineered EVs such as stability, tropism and release kinetics might also require further improvement before EVs with engineered membrane or cargo could be used in the clinic. As discussed earlier, commercial scale manufacture of engineered EVs, which could possibly be achieved in the future using high‐throughput approaches, such as 3D‐bioprinting technology (Di Marzio et al., 2020; Maiullari et al., 2021; Wlodarczyk‐Biegun & Del Campo, 2017), would require adequate understanding of necessary process controls to ensure EV consistency. As shown in this workshop, EVs with engineered therapeutic cargo or membrane can be either derived from primary cells‐ or synthetic nanoparticles with synthetic or cell‐extracted membranes (Fang et al., 2018; Gao, Chu et al., 2016; Witwer & Wolfram, 2021). Currently, the pros versus cons of using one type over the other remain poorly understood. The advantage of cell‐derived EVs is their ability to cross tissue barriers and deliver functional cargo, while targeted delivery of nanoparticles to specific organs other than the liver is still challenging (Akinc et al., 2010). However, the well‐characterized formulation, efficient loading of diverse cargo, feasibility of surface/content modifications and the scalability to fabricate make synthetic nanoparticles, such as LNPs, promising drug delivery vehicles for HLBS disease therapies. Also, the potential immunogenicity and risk of developing graft‐versus‐host‐disease (GVHD) with administration of cell‐derived EVs was also highlighted as a major concern by the expert panel. Certain key parameters, such as the criteria for choosing a potential human donor for generating cell‐derived EVs and whether source material should be restricted to autologous cells remain poorly understood. More preclinical studies in animal models would help to determine which EVs would be appropriate for carrying a specific therapeutic cargo for delivery to a specific tissue in a specific HLBS disease condition. Regardless of the type of EV, optimizing the quantity of therapeutic cargo in EVs to attain the desired biological effect was identified as a major challenge by the expert panel; this will require more analytical and preclinical studies in the future. Fortunately, similar dosing strategy challenges have been addressed in the past in the field of LNP‐based targeted drug‐delivery (Dawidczyk et al., 2014; van der Koog et al., 2022). The field of therapeutic EV‐engineering could possibly benefit from the lessons learned in the field of drug delivery, such as the use of in vivo, high throughput technology to screen for engineered EVs with optimal attributes. As shown in this workshop, EV engineering for HLBS diseases therapy can also benefit from the knowledge of the endogenous biological mechanisms underlying EV generation by cells, such as stem cells or tumour cells, uptake of these EVs by other cells in vivo and how these EVs contribute to cell‐cell communication in the microenvironment of bone marrow, tumour or other tissues (van der Pol et al., 2016; van Niel et al., 2018; Vats et al., 2020). Evidence shared in this workshop also suggested that genetically or biochemically modified cells or tissues might be potential sources to generate therapeutic EVs. Similarly, biochemical modification of the microenvironment in the target tissue may also facilitate the delivery and uptake of the therapeutic EVs by the target tissue. However, these approaches are associated with major technological limitations and more in vivo preclinical studies in rodents, as well as large animal models such as pigs or primates, would be needed to validate them.

Improved understanding of the biology of EV biogenesis, targeting, membrane docking, tethering, followed by uptake by target cells would also facilitate the use of the EVs for novel diagnostics and prognostics in HLBS diseases. Development of novel technologies, such as nanoflow cytometry or microfluidic platforms capable of detecting rare populations of cell‐specific EVs or certain miRNA enriched‐EVs in the plasma, might improve clinical outcomes and reduce health care costs by increasing the specificity for diagnosis of HLBS diseases at early time points (Reategui et al., 2018; Tian et al., 2020). Also, preclinical studies in rodent models of HLBS diseases could identify alterations in subpopulations of cell‐specific EVs, the cargo of such cell‐specific EVs and the function of these EVs using novel analytical approaches to guide the diagnosis of HLBS diseases in future clinical studies. Several key areas of research and development were identified by the expert panel to facilitate EV‐based diagnostics of HLBS diseases, such as the development of customized kits to enable EV biomarker detection in plasma samples without the need for sample processing, avoiding cryopreservation, validation of reliable housekeeping gene(s) for RNA sequencing studies with EVs, and encouraging the use of standardized reference materials.

In conclusion, several key areas warranting potential future investigation were identified in this NHLBI workshop to improve the therapeutic use of EVs in HLBS diseases. The current state‐of ‐the‐art and major limitations associated with generating therapeutic EVs by either engineering the EV membrane or EV cargo were identified (summary of pros vs. cons shown in Table 2). Opportunities were discussed to develop novel analytical approaches that could improve the reproducibility, efficacy and commercial scale production of therapeutic EVs for clinical use. Lessons were drawn from the knowledge of the fundamental biology underlying EV‐biogenesis by primary cells, targeting to specific cells and tissues, EV uptake by the target cells and opportunities were suggested regarding how this information might be harnessed to further improve engineering of the therapeutic EVs. Lastly, areas of potential future research were identified to enable the use of EVs in the diagnosis and prognostics of HLBS diseases.

**TABLE 2:** Pros versus cons of the EV engineering approaches

EV engineering approach			Pros	Cons
Endogenous engineering of EV‐secreting cells	Cell conditioning		Enrichment of the endogenous cargo of interest	Contamination by other conditioning‐responsive cargoes
	Passive loading		Convenient loading procedures	Low EV loading efficiency
	Genetic manipulation		Versatile strategies enabling the loading of almost all genes‐of‐interest into EVs	Gene manipulation may change the status of cells and the following secretion and cargo of EVs
			Feasibility of scaled‐up manufacturing	
Exogenous modification of isolated EVs	Passive loading		Convenient loading procedures	Low loading efficiency and risk of contamination by unwanted cargoes
	Encapsulation of nanoparticles		Drug loading versatility of nanoparticles	Challenges of identifying ideal encapsulation method
	Active loading	Electroporation	Diverse loading compounds and high loading efficiency	Risk of altering the physicochemical, morphological, and biophysical characteristics of EVs and inducing EV aggregation Risk of contamination by transfection reagents
		Sonication		
		Freeze‐thaw cycles		
		Surfactant treatment		
		Chemical transfection		

## AUTHOR CONTRIBUTIONS

Prithu Sundd and Guoping Li wrote the manuscript in consultation with other coauthors. Stephen Y. Chan, Saumya Das and Guofei Zhou contributed to the writing and editing of the manuscript. Renee Wong, Margaret J. OchocinskaMalcolm Moos, Martha S. Lundberg, Tianji Chen, James Dahlman, Lola Eniola‐Adefeso, Ionita C. Ghiran, Peter Kurre, Wilbur A. Lam, Jennifer K. Lang, Eduardo Marbán, Pilar Martín, Stefan Momma, Malcolm Moos, Deborah J. Nelson, Robert L. Raffai, Xi Ren, Joost P.G. Sluijter, Shannon L. Stott, Gordana Vunjak‐Novakovic, Nykia D. Walker, Zhenjia Wang, Kenneth W. Witwer and Phillip C. Yang contributed to editing of the manuscript.

## CONFLICT OF INTEREST

Prithu Sundd received funding as a part of sponsored research agreements with CSL Behring Inc., IHP Therapeutics and Novartis Pharmaceuticals Corporation. He is also the recipient of Bayer Hemophilia Award and has filed patent application targeting Gasdermin‐D to prevent lung injury in Sickle Cell Disease. Stephen Y. Chan has served as a consultant for Acceleron Pharma and United Therapeutics. He is a director, officer and shareholder in Synhale Therapeutics and has held research grants from Actelion, Bayer and Pfizer. Stephen Y. Chan has also filed patent applications regarding the targeting of metabolism in pulmonary hypertension. Kenneth W. Witwer is an officer of the International Society for Extracellular Vesicles (ISEV), has served as an advisor for Neurodex and ShiftBio and an ad hoc consultant with NeuroTrauma Sciences, Kineticos, King Abdulaziz University and Burst Biologics, and has held research grants from AgriSciX, Yuvan Research, and Ionis Pharmaceuticals. The remaining authors declare no competing financial interests.
