# Correction to “Current challenges and future directions for engineering extracellular vesicles for heart, lung, blood and sleep diseases”

**DOI:** 10.1002/jev2.12314

**Published:** 2023-03-22

Li, G., Chen, T., Dahlman, J., Eniola‐Adefeso, L., Ghiran, I. C., Kurre, P., Lam, W. A., Lang, J. K., Marbán, E., Martín, P., Momma, S., Moos, M., Nelson, D. J., Raffai, R. L., Ren, X., Sluijter, J. P. G., Stott, S. L., Vunjak‐Novakovic, G., Walker, N. D., … Sundd, P. (2023). Current challenges and future directions for engineering extracellular vesicles for heart, lung, blood and sleep diseases. *Journal of Extracellular Vesicles*, 12, e12305. https://doi.org/10.1002/jev2.12305

In the originally published article, author Saumya Das was incorrectly credited as Saumta Das. This has been corrected in the online version of the article.

We apologize for this error.

